# The Prevalence and Stability of Sleep-Wake Disturbance and Fatigue throughout the First Year after Mild Traumatic Brain Injury

**DOI:** 10.1089/neu.2019.6898

**Published:** 2020-11-06

**Authors:** Simen Berg Saksvik, Migle Karaliute, Håvard Kallestad, Turid Follestad, Robert Asarnow, Anne Vik, Asta Kristine Håberg, Toril Skandsen, Alexander Olsen

**Affiliations:** ^1^ Department of Psychology, Norwegian University of Science and Technology, Trondheim, Norway.; ^2^ Department of Physical Medicine and Rehabilitation, St. Olavs Hospital, Trondheim University Hospital, Trondheim, Norway.; ^3^ Department of Mental Health, Norwegian University of Science and Technology, Trondheim, Norway.; ^4^ Department of Public Health and Nursing, Norwegian University of Science and Technology, Trondheim, Norway.; ^5^ Department of Psychology, University of California, Los Angeles, Los Angeles, California, USA.; ^6^ Department of Psychiatry, University of California, Los Angeles, Los Angeles, California, USA.; ^7^ Department of Neuromedicine and Movement Science, Norwegian University of Science and Technology, Trondheim, Norway.; ^8^ Department of Neurosurgery, St. Olavs Hospital, Trondheim University Hospital, Trondheim, Norway.; ^9^ Department of Radiology and Nuclear Medicine, St. Olavs Hospital, Trondheim University Hospital, Trondheim, Norway.

**Keywords:** fatigue, hypersomnia, insomnia, mild traumatic brain injury, sleep-wake disturbances

## Abstract

In this prospective, longitudinal study, we aimed to determine the prevalence and stability of sleep-wake disturbance (SWD) and fatigue in a large representative sample of patients (Trondheim mild traumatic brain injury [mTBI] follow-up study). We included 378 patients with mTBI (age 16–60), 82 matched trauma controls with orthopedic injuries, and 83 matched community controls. Increased sleep need, poor sleep quality, excessive daytime sleepiness, and fatigue were assessed at 2 weeks, 3 months, and 12 months after injury. Mixed logistic regression models were used to evaluate clinically relevant group differences longitudinally. Prevalence of increased sleep need, poor sleep quality, and fatigue was significantly higher in patients with mTBI than in both trauma controls and community controls at all time points. More patients with mTBI reported problems with excessive daytime sleepiness compared to trauma controls, but not community controls, at all time points. Patients with complicated mTBI (intracranial findings on computed tomography or magnetic resonance imaging) had more fatigue problems compared to those with uncomplicated mTBI, at all three time points. In patients with mTBI who experienced SWDs and fatigue 2 weeks after injury, around half *still* had problems at 3 months and approximately one third at 12 months. Interestingly, we observed limited overlap between the different symptom measures; a large number of patients reported one specific problem with SWD or fatigue rather than several problems. In conclusion, our results provide strong evidence that mTBI contributes significantly to the development and maintenance of SWDs and fatigue.

## Introduction

Sleep-wake disturbance (SWD) and fatigue are common after mild (mTBI) traumatic brain injury (TBI) and have been linked to a range of adverse consequences, including reduced cognitive functioning,^1,2^ emotional distress,^1,3^ and reduced quality of life.^4^ Insomnia and hypersomnolence disorder are the most common SWDs after TBI of all severities.^5^ In the *Diagnostic and Statistical Manual of Mental Disorders*, fifth edition (DSM-5), insomnia disorder is characterized by poor sleep quality, and hypersomnolence disorder by excessive sleep quantity and daytime sleepiness.^6^ In a meta-analysis, 50% of TBI patients across all severities experienced some form of SWD after injury.^7^ Both insomnia and hypersomnolence disorder are accompanied by daytime distress, often in the form of fatigue.^6^

A recent systematic literature review indicates that between 17% and 47% of patients with mTBI may experience fatigue during the first 3 months after injury.^8^ Problems with increased sleep need, poor sleep quality, excessive daytime sleepiness, and fatigue appear to decrease during the first month,^1,9,10^ but stabilize at higher levels than before the injury between 3 and 6 months after mTBI.^1,10^ Because SWD and fatigue are prevalent in the general population^11–13^ and after traumatic injuries not involving the head,^14^ it is important to carefully consider the choice of comparison groups included in studies investigating such problems after mTBI.^15,16^

Factors specific to mTBI that can be involved in the development or maintenance of SWD and fatigue include primary damage to the brain as a direct consequence of the injury or secondary to, for example, mood or lifestyle changes after mTBI.^17^ Pre-injury factors, such as personality factors or a history of psychiatric problems, may also affect SWD and fatigue after mTBI.^18^ General injury-related factors,^14,19^ such as medication use^20^ and injury-related pain or stress,^21,22^ might lead to SWD and fatigue, but are also present after injuries not involving the head (e.g., orthopedic injuries). Moreover, SWD has increased in the general population, likely because of higher work demands, increased shift and night work, and more excessive environmental stimuli (e.g., computer/cell-phone screen time).^11^

In previous longitudinal studies with control groups, patients with mTBI and previous psychiatric problems have been excluded,^23–25^ which might have biased these studies toward more homogeneous mTBI samples, not generalizable to the heterogenetic mTBI population.^26^ An additional source of bias is that the majority of patients with mTBI who are treated outside of hospitals^27^ have not been included in most previous studies.^28^ Only one population-based study investigating SWD after mTBI has recruited patients in the primary healthcare setting, but this study did not include any control groups.^1^ Earlier studies have also typically aggregated SWD and fatigue-related findings across all TBI severities. Studies focusing on well-characterized mTBI patients are therefore needed.^29^

Most studies investigating SWD after mTBI have assessed only one facet of sleep, with poor sleep quality being the most frequently investigated symptom.^30^ Information about how increased sleep need, poor sleep quality, excessive daytime sleepiness, and fatigue coexist is important for understanding potentially shared and distinct etiology,^31^ as well as different phenotypes^5^ after mTBI.

Here, we investigated a broad range of SWDs and fatigue in a large representative sample of patients with mTBI.^32^ Our main aim was to evaluate the prevalence and stability of SWD and fatigue throughout the first year after mTBI. To extend and substantiate earlier studies, we compared patients with mTBI to two control groups (patients with orthopedic injuries and community controls) throughout the first year after injury on measures of increased sleep need, poor sleep quality, excessive daytime sleepiness, and fatigue. To substantiate our findings, we evaluated the overlap between different types of SWD and fatigue problems. Finally, to further evaluate possible associations with brain-injury–specific factors, we investigated whether there were any differences between patients with complicated mTBI (intracranial findings on computed tomography [CT] or magnetic resonance imaging [MRI]) and patients with uncomplicated mTBI.

## Methods

### Participants

In this longitudinal cohort study,^32^ we recruited patients with mTBI prospectively from two emergency departments (EDs); the level 1 trauma center at St. Olavs Hospital and the general-practitioner–run Trondheim municipal clinic. These two EDs evaluate the vast majority of acute mTBI in Trondheim and adjacent regions. Both EDs are part of the public healthcare system in Norway, located at the university hospital campus, and use the same CT service. These two EDs serve ∼230,000 residents in Trondheim and adjacent regions in addition to ∼18,000 students (who are officially registered as residents in other parts of the country). The inclusion period was between April 1, 2014 and December 5, 2015.

Participants were included if they were between 16 and 60 years of age and had sustained an mTBI according to the World Health Organization criteria.^33^ Patients had to have a witnessed loss of consciousness (LOC) <30 min, post-traumatic amnesia (PTA) <24 h, or evident lesions on CT, in addition to a Glasgow Coma Scale (GCS) score between 13 and 15. Exclusion criteria were: 1) non-fluency in Norwegian or residence in another country; 2) any *severe* ongoing alcohol/drug abuse, psychiatric or somatic disease, or condition that would complicate follow-up; 3) any previous complicated mild, moderate, or severe TBI, stroke, or other acquired brain injuries; 4) severe concurrent multi-trauma, such as spinal cord injury, internal bleeding, or severe fractures; and 5) presentation to the ED >72 h of injury. A main aim of the overarching cohort study was to evaluate neuroimaging findings from CT and MRI.^32^ Patients with previous *complicated* mTBI were excluded to reduce the likelihood of including patients with *pre-existing* gross brain pathology. Apart from this, we aimed to be as inclusive as possible to optimize the clinical representativeness in the sample.

Trauma controls with orthopedic injuries (e.g., sprains or fractures) were included between April 1, 2015 and December 1, 2017 from the same two clinics as the patients with mTBI. Trauma controls were identified by screening patient lists at the ED at the municipal clinic and lists of referrals to the hospital. They were evaluated for inclusion if they were between 16 and 60 years of age and had sustained an orthopedic injury. They could not have any evidence of head or neck trauma, polytrauma, or injury to their dominant upper extremity. Other exclusion criteria were identical to those applied for the mTBI group.

Community controls, between 16 and 60 years of age, were included from a convenience sample recruited from employees and students at the university hospital as well as from friends and family of the patients with mTBI, or from employees and students at the university hospital. Exclusion criteria were the same as for the patients with mTBI, with the additional criteria that the community controls did not receive any ongoing treatment for psychiatric disorders. Participants in the community control group were matched at group level to the mTBI group in terms of age, sex, and length of education in years, whereas participants in the trauma control group were matched at group level to the mTBI patients in terms of age and sex.

### Procedure

**FIG. 1.**
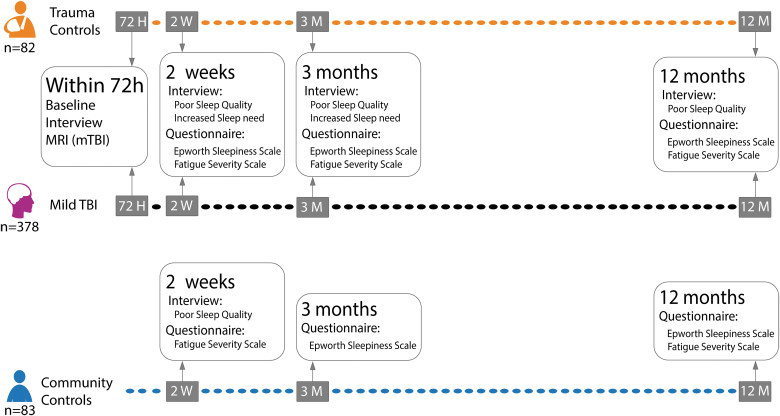
Timeline of follow-up. MRI, magnetic resonance imaging; mTBI, mild traumatic brain injury; TBI, traumatic brain injury.

Detailed procedures for recruitment to the study have been described elsewhere.^32^ Patients with mTBI were assessed at four time points (Fig. 1). Within 72 h of their injury, the patients with mTBI completed a structured interview containing questions related to injury characteristics, demographics, and premorbid health problems. Clinical MRI (3 Tesla) was performed within 72 h of injury. At 2 weeks, 3 months, and 12 months after injury, patients with mTBI completed a structured interview and questionnaires. Trauma controls underwent the same procedures as patients with mTBI, except MRI. Community controls also underwent the same procedures as patients with mTBI, excluding the first visit (within 72 h of injury).

A subgroup of patients with mTBI did not meet at the hospital, but answered interviews over the telephone and sent completed questionnaires by mail. Participants who met at the hospital for their assessments received a gift certificate (∼EUR 54) for each attendance. All participants gave their informed consent. The project was performed according to the Helsinki Declaration and was approved by the Regional Committee for Medical and Health Research Ethics (REK) in Norway (REK 2013/754).

### Measures

#### Brain imaging

Non-contrast head CT was performed on a Siemens Somatom Sensation 64-row scanner (Siemens Healthineers, Erlangen, Germany) in 299 of the patients as a part of routine clinical assessment. In addition, brain MRI scans were acquired on a 3 Tesla Siemens Skyra System (32-channel head coil; Siemens Healthineers) within 72 h of injury for 198 patients with mTBI. The following sequences were included: three-dimensional T_1_, T_2_, susceptibility-weighted imaging, diffusion-weighted imaging (DWI), and fluid-attenuated inversion recovery. All CT and MRI scans were read by experienced radiologists,^34^ and patients with mTBI and intracranial traumatic findings on CT and/or MRI were considered to have complicated mTBI.

#### Structured interviews

In the first interview, completed within 72 h of injury, participants were asked to provide information about cause of injury, LOC, PTA, pre- and post-injury medication use, pain, previous health problems, and previous uncomplicated mTBI. Information about previous uncomplicated mTBI was based on self-report as recommended in other studies.^35^ Three subsequent interviews were performed at 2 weeks, 3 months, and 12 months after injury to assess post-injury problems and functioning.

##### Pre-injury psychiatric disorders

A structured interview during the first study visit was performed to assess previous psychiatric history. Participants were asked to report and describe any previous psychiatric disorders. Participants that reported psychiatric disorders were further asked to describe the onset and duration (if relevant) of the disorder and to what degree the disorder affected their daily life. Based on information from the structured interview, patients were categorized as having a pre-injury psychiatric disorder or not.

##### Increased sleep need

Participants were asked to report their average total sleep time during the 2 weeks before inclusion in the study (i.e., before the injury for the patient groups). Second, participants were asked whether they experienced any increased sleep need after injury. Participants who answered *yes* to this question were further asked to report the duration (in days) of this increased sleep. Finally, participants were asked to report their average total sleep time in the period they experienced this increased sleep need. Increased total sleep time of >1 h is commonly used as a measure of increased sleep need in TBI research.^36–38^ We therefore classified patients with mTBI into an increased sleep need group that reported to sleep >1 h longer after injury than their average total sleep time before the injury and a group that did not experience increased sleep need. We applied the same dichotomization for trauma controls.

##### Poor sleep quality

Three items from the Insomnia Severity Index^39^ were included to investigate symptoms of insomnia. These items were selected based on being direct measures of insomnia according to the diagnostic manual DSM-5,^6^ namely subjective difficulties falling asleep (sleep onset insomnia), difficulties staying asleep (maintenance insomnia), and unwanted early morning awakenings (terminal insomnia). Participants were asked to rate how much of a problem each item had been during the last 2 weeks using a 5-point Likert-type scale, 0 to 4 (0 = No problem, 1 = A mild problem, 2 = A moderate problem, 3 = A severe problem, and 4 = A very severe problem). Participants were classified as poor sleepers if they reported at least severe difficulties on one of these three items, which is in line with criterion A of Insomnia Disorder in DSM-5.^6^

#### Questionnaires

##### Pain map with Numeric Rating Scale

Participants were asked whether they experienced any pain beyond that of everyday mundane pain after their injury. They were then asked to rate the pain intensity, using an 11-point Numeric Rating Scale (NRS) from 0 to 10, and to indicate the location of their pain using a body map.

##### Excessive daytime sleepiness: Epworth Sleepiness Scale

The Epworth Sleepiness Scale (ESS) consists of eight items measuring self-reported excessive daytime sleepiness.^40^ The eight items represent real-life situations where the participants must rate their chance of dozing off using a 4-point scale from 0 to 3. Higher scores indicate higher chance of dozing off. Total score indicates the extent of self-reported sleep propensity. The Norwegian version of the ESS has high validity^12^ and high reliability.^41^ According to studies comparing the ESS to objectively measured sleepiness,^42^ we applied a cutoff of 13 and above.

##### Fatigue: Fatigue Severity Scale

The Fatigue Severity Scale (FSS) measures feelings of fatigue, with nine items rated on a 7-point scale from 1 to 7.^43^ Higher total scores indicate higher levels of fatigue. A total mean score of 4 was originally defined as clinically significant fatigue.^43^ The Norwegian version of FSS has been shown to have satisfactory psychometric properties.^44^ According to a validation study in a Norwegian sample, we applied a cutoff of 5 and above.^44^

Patients with mild TBI and trauma controls completed all the interviews and questionnaires at all time points. Community controls did not answer the questions about increased sleep need, but answered poor sleep quality questions at the 2 weeks after injury time point, excessive daytime sleepiness at the 3 and 12 months after injury time points, and fatigue at the 2 weeks and 12 months after injury time points.

### Statistical analysis

We performed attrition analyses and investigated differences between participants with missing data and participants with complete data on demographic variables, using chi-square tests and independent-sample *t*-tests to evaluate the pattern of missing data. Based on these attrition analyses, a missing at random, but not missing *completely* at random, assumption was most reasonable.^45^

Chi-square tests and independent-sample *t*-tests were used to investigate whether the control groups were successfully matched on a group level with the mTBI group on variables of sex, age, and total years of completed education. We also performed chi-square tests, independent-sample *t*-tests, Mann-Whitney U tests, and Kruskal-Wallis tests to evaluate other group differences on key demographic- and injury-related variables.

We used mixed logistic regression, a generalized linear mixed model for binary outcomes, to investigate differences in increased sleep need, poor sleep quality, excessive daytime sleepiness, and fatigue over time between patients with mTBI and trauma controls. The models included the fixed factors time, group, and their interaction and a random intercept on logit scale to account for within-subject dependencies. Additional mixed logistic regression models, based on data from time points where community controls had available data, were used to compare patients with mTBI to trauma controls as well as community controls. Data for community controls were available for fatigue at 2 weeks and 12 months after injury and excessive daytime sleepiness at 3 and 12 months after injury. A logistic regression model was used to compare patients with mTBI to trauma controls and community controls on poor sleep quality 2 weeks after injury (the time point at which community controls had available data). For all analyses performed, *p* values <0.05 were considered statistically significant.

To investigate the effects of complicated mTBI (intracranial lesions visible on MRI or CT) on SWD and fatigue, we performed mixed logistic regression models to investigate differences in SWD and fatigue between patients with complicated mTBI, uncomplicated mTBI, and trauma controls. Community controls were not included in the models comparing patients with complicated and uncomplicated mTBI because they did not have data on all time points. We controlled for age, sex, and pre-injury psychiatric disorders in all models.

To investigate the overlap between increased sleep need, poor sleep quality, excessive daytime sleepiness, and fatigue, we performed a frequency count to investigate the degree of overlap between these four problems at the respective time points.

The data handling, attrition analyses, and investigation of whether the groups were successfully matched were performed in SPSS software (version 25; SPSS, Inc., Chicago, IL). The mixed logistic regression analyses as well as logistic regression analysis were performed in STATA software (version 15; StataCorp LP, College Station, TX), and overlap figures were created with the R package, eulerr (R Foundation for Statistical Computing, Vienna, Austria).^46^

## Results

We identified a total of 624 mTBI cases from screening lists of CT referrals (Fig. 1). Of these, a total of 299 patients with mTBI met the inclusion criteria and agreed to participate in the study. An additional 79 patients with mTBI without CT scans were recruited from the EDs, by screening patient lists. Accordingly, a total of 378 patients with mTBI, 82 trauma controls, and 83 community controls were included in the study (Fig. 1). Key demographic and clinical variables are presented in Table 1. Patients with mTBI, trauma controls, and community controls were successfully matched and did not differ significantly on the variables age, sex, and years of completed education. Patients with mTBI who completed their assessments at the hospital and those who completed these at home did not differ significantly on the variables age (mean [M] = 32.5, standard deviation [SD] = 13,1 vs. M = 29.9, SD = 12.7, *p* = 0.054), sex (χ^2^_(1)_ = 1.04, *p* = 0.306) and years of completed education (M = 13.9, SD = 2.50 vs. M = 13.4, SD = 2.4, *p* = 0.053).

**Table 1.** Participant Characteristics

Variable	Mild TBI patients *n* = 378	Trauma controls *n* = 82	Community controls *n* = 83
Sex			
Men	247 (65.3%)	51 (62.2%)	50 (60.2%)
Women	131 (34.7%)	31(37.8%)	33 (39.8%)
Age			
Mean (SD)	31.2 (12.9)	32.6 (13.0)	33.1 (13.0)
Education, years			
Mean (SD)	13.6 (2.5)	14.3 (2.6)	14,0 (2.4)
Employment status			
Employed	215 (56.9%)	48 (58.5%)	52 (62.7%)
Unemployed	24 (6.3%)	2 (2.4%)	2 (2.4%)
Student	138 (36.5%)	31 (37.8%)	27 (32.5%)
Missing	1 (0.3%)	1 (1.2%)	2 (2.4%)
GCS score			
13	5 (1.3%)		NA	NA
14	57 (15.1%)		NA	NA
15	277 (73.3%)		NA	NA
Unknown	39 (10.3%)		NA	NA
PTA duration				
< 1 hour	271 (71.7%)		NA	NA
1 hour–24 hours	107 (28.3%)		NA	NA
LOC duration				
No LOC	67 (17.7%)		NA	NA
< 5 min	157 (41.5%)		NA	NA
5–15 min	15 (4.0%)		NA	NA
15–30 min	1 (0.3%)		NA	NA
Difficult to assess	6 (1.6%)		NA	NA
Unknown	132 (34.9%)		NA	NA
Injury mechanism				
Fall	135 (35.7%)	26 (31.7%)	NA	
Motor Vehicle accident	43 (11.4%)	3 (3.7%)	NA	
Bicycle accident	58 (15.3%)	7 (8.5%)	NA	
Violence	65 (17.2%)	1 (1.2%)	NA	
Sports accident	54 (14.3%)	30 (36.6%)	NA	
Hit object	17 (4.5%)	6 (7.3%)	NA	
Other^*^	3 (0.8%)	9 (11.0%)	NA	
Unknown	3 (0.8%)	0 (0%)	NA	
Surgery (TC)				
(%yes/no)		NA	25/75	NA
Prior Uncomplicated mild TBI				
No prior mTBI	292 (77.2%)	75 (91.5%)	73 (88.0%)	
1	65 (17.2%)	4 (4.9%)	8 (9.6%)	
2	15 (4.0%)	2 (2.4%)	0	
3	1 (0,3%%		0	0
4	1 (0,3%)		0	0
Missing	4 (1.1)	1 (1.2%)	2 (2.4)	
Mean years since last prior mTBI (SD)	12.2 (10.3)	14.6 (9.22)	23.5(13.6)	
CT				
Normal	252 (66.7%)		NA	NA
No CT	79 (20.9%)		NA	NA
Facial Fracture	18 (4.8%)		NA	NA
Cranial Fracture	5 (1.3%)		NA	NA
Intracranial lesions	11 (2.9%)		NA	NA
Fracture and intracranial lesions	11 (2.9%)		NA	NA
Other	2 (0.5%)		NA	NA
MRI (within 72h of injury)				
Normal	172 (45.5%)		NA	NA
Extracranial findings	3 (0.8%)		NA	NA
Intracranial findings	23 (6.1%)		NA	NA
Not performed	180 (47.6%)		NA	NA
Prescribed psychotropic medication before injury/study inclusion				
Yes	25 (6.6%)	7 (8.5%)	2 (2.4%)	
No/No answer	353 (93.4%)	75 (91.5%)	81 (97.6%)	
Prescribed medication after injury				
Yes	39 (10%)	22 (27%)	NA	
No/No answer	339 (90%)	60 (73%)	NA	
Average total sleep time the two weeks before injury/study inclusion				
Mean (SD)	7.09 (1.11)	7.18 (1.03)	6.90 (0.92)	
Poor sleep quality the two weeks before injury/study inclusion				
Yes	25 (6.6%)	5 (6.1%)	2 (2.4%)	
No	346 (91.5%)	76 (92.7%)	78 (94.0%)	
Missing	7 (1.9%)	1 (1.2%)	3 (3.6%)	
Prior Psychiatric Disorders				
No psychiatric history	316 (83.6%)	72 (87.8%)	69 (83.1%)	
Depression	22 (5.8%)	6 (7.3%)	4 (4.8%)	
Anxiety	17 (4.5%)	2 (2.4%)	1 (1.2%)	
Other^**^	16 (4.2%)	0 (0%)	1 (1.2%)	
Missing	7 (1.8%)	2 (2.4%)	8 (9.6%)	

^*^ Other injuries in the trauma control group include sharp injuries, such as cuts.

^**^ Other psychiatric disorders include disorders that few individuals reported such as eating disorders

GCS: Glasgow Coma Scale, PTA: Post Traumatic Amnesia, LOC: Loss of consciousness, CT: Computed Tomography, MRI: Magnetic Resonance Imaging, OCD: Obsessive Compulsive Disorder; NA, not applicable.

The majority of patients with mTBI had a PTA duration <1 h (72%), LOC duration <5 min (42%), and a GCS score of 15 (73%). Sixty-five percent of patients with mTBI were men. Most of the sample was employed (58%) or students (36%). Most patients with mTBI had sustained their first-ever mTBI (77%). In total, 332 patients with mTBI had a head CT scan, brain MRI scan, or both CT and MRI; of these, 9% (*n* = 31) had complicated mTBI (intracranial pathology on CT or MRI). Proportions of different causes of injury were significantly different between patients with mTBI and trauma controls (*p* < 0.001; Table 1). Falls were the most common cause of injury for both patients with mTBI (36%) and trauma controls (32%). More patients with mTBI than trauma controls were injured because of violence (17% vs. 1%) and motor vehicle accidents (11% vs. 4%), but trauma controls were more often injured because of sports accidents than patients with mTBI (37% vs. 14%). There were no statistically significant differences between patients with mTBI and the two control groups in occupational status (*p* = 0.379; Table 1), or between patients with mTBI and trauma controls in time to return to work at any of the time points after injury (Table 2).

**Table 2.** Time Related Variables

Variable	Mild TBI patients *n* = 378	Trauma controls *n* = 82	Community controls *n* = 83	*p*-value
	n (%)	n (%)	n (%)	
Return to work/studies at 2 weeks				
No sick leave	147 (38.8%)	32 (39.0%)	NA	p = 0.122
Returned to work/studies	93 (24.6%)	14 (17.1%)	NA	
Not returned to work/studies	91 (24.1%)	27 (32.9%)	NA	
Returned part-time	11 (2.9%)	5 (6.1%)	NA	
Unemployed before injury	24 (6.3%)	2 (2.4%)	NA	
Missing	12 (3.2%)	2 (2.4%)	NA	
Return to work/studies at 3 months				
No sick leave	126 (33.3%)	29 (35.4%)	NA	p = 0.601
Returned to work/studies	165 (43.6%)	33 (40.2%)	NA	
Not returned to work/studies	26 (6.9%)	8 (9.8%)	NA	
Returned part-time	15 (4.0%)	3 (3.7%)	NA	
Unemployed before injury	24 (6.4%)	2 (2.4%)	NA	
Missing	22 (5.8%)	7 (8.5%)	NA	
Return to work/studies at 12 months				
No sick leave	93 (24.6%)	27 (32.9%)	NA	p = 0.202
Returned to work/studies	159 (42.1%)	39 (47.6%)	NA	
Not returned to work/studies	23 (6.1%)	3 (3.7%)	NA	
Returned part-time	5 (1.3%)	0 (0%)	NA	
Unemployed before injury	24 (6.3%)	2 (2.4%)	NA	
Missing	74 (19.6%)	11 (13.4%)	NA	
Pain location 2 weeks				
Head, Neck or face	105 (39.2%)	8 (10.7%)	6 (8%)	p < 0.001
Other parts of the body	33 (12.3%)	41 (54.7%)	15 (20%)	
No pain	130 (48.5%)	26 (34.7%)	55 (72%)	
Pain location at 3 months				
Head, Neck or face	53 (22.6%)	6 (8.0%)	NA	p < 0.001
Other parts of the body	25 (10.7%)	24 (32.0%)	NA	
No pain	156 (66.7%)	45 (60.0%)	NA	
Pain location at 12 months				
Head, Neck or face	48 (20.1%)	4 (6.1%)	NA	p < 0.001
Other parts of the body	20 (8.4%)	18 (27.3%)	NA	
No pain	171 (71.5%)	44 (66.7%)	NA	
		Median (range)	Median (range)	Median (range)	
Pain intensity (NRS 0–11) at 2 weeks	1 (10)	2 (9)	0 (6)	p < 0.001	
Pain intensity (NRS 0–11) at 3 months	0 (8)	0 (7)	NA	p = 0.375	
Pain intensity (NRS 0–11) at 12 months	0 (10)	0 (8)	NA	p = 0.745	

Chi squre tests were used to investigate group differences in return to work and pain location 2 weeks, 3 months and 12 months after injury. Kruskal-Wallis test was used to investigate group differences in pain intensity at 2 weeks. Mann-Whitney U tests were performed to investigate which groups were statistically significant from one another in pain intensity at 2 weeks. These tests showed that patients with mTBI (U = 7211, p < 0.001, r = 0.22) and trauma controls (U = 1589, p < 0.001, r = 0.61) had significantly higher pain intensity compared to community controls. Patients with mTBI did not differ significantly from trauma controls in pain intensity 2 weeks after injury (U = 8821, p = 0.090, r = .0.09. Mann-Whitney U tests were also used to investigate differences between patients with mTBI and trauma controls in pain intensity 3 months and 12 months after injury.

NRS, Numeric Rating Scale.

We observed no significant differences between patients with mTBI and the two control groups in self-reported total sleep time the 2 weeks before study inclusion (*p* = 0.160; Table 1). At 2 weeks, the time point where community controls had available data, both patients with mTBI and trauma controls reported significantly higher pain levels than community controls (Table 2). Patients with mTBI and trauma controls did not differ significantly in levels of pain intensity at 2 weeks, 3 months, or 12 months. Throughout all time points, most patients with mTBI who reported pain had pain in their head, face, or neck region, whereas most trauma controls and community controls who reported pain had pain in other parts of the body (Table 2).

**FIG. 2.**
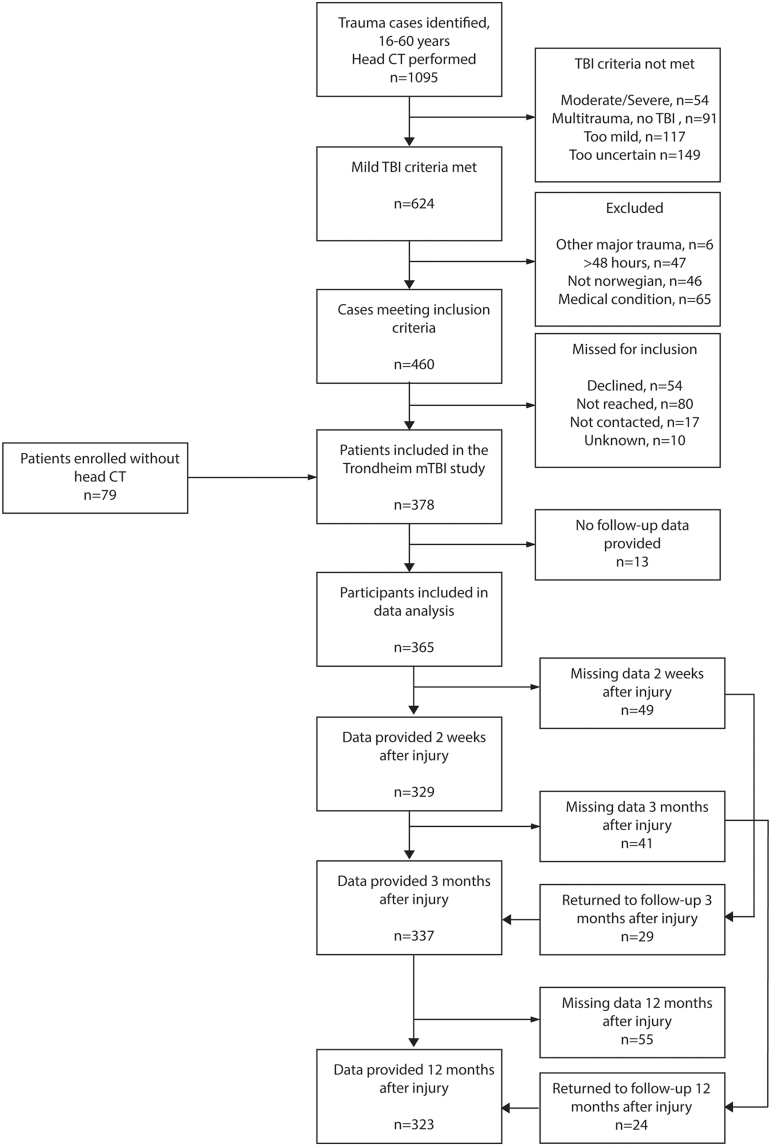
Flowchart of follow-up, patients with mTBI. CT, computed tomography; mTBI, mild traumatic brain injury; TBI, traumatic brain injury.

Of the 378 included patients with mTBI, 333 (88%) completed the 3-month interview, and 321 (85%) completed the 12-month interview. The 3-month questionnaire was completed by 235 patients (62%) and the 12-month questionnaire by 239 patients (63%). Most patients had more than one follow-up time point with interview data (*n* = 363; 96%) and questionnaire data (*n* = 298; 78%). In total, 365 patients with mTBI (97%; Fig. 2), 79 trauma controls (96%), and 77 community controls (93%) had at least one follow-up time point after baseline. In total, 56 patients with mTBI (15%), 11 trauma controls (13%), and 15 community controls (18%) were lost to follow-up.

### The prevalence of sleep-wake disturbance and fatigue

**FIG. 3.**
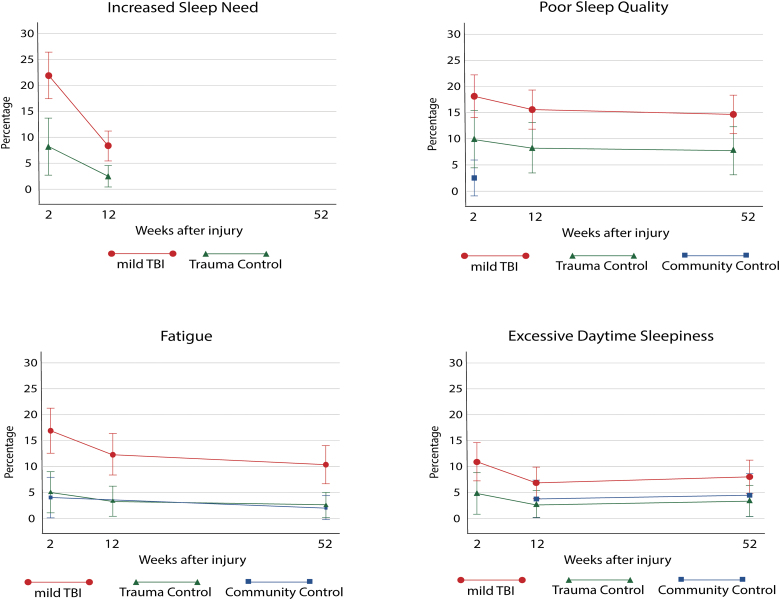
Estimated proportion of patients with mTBI, trauma controls, community controls with sleep-wake disturbances, and fatigue over the course of 1 year after injury. mTBI, mild traumatic brain injury.

Patients with mTBI consistently reported higher rates of having problems with sleep need, poor sleep quality, excessive daytime sleepiness, and fatigue than both control groups. Results from mixed logistic regression models are presented in Table 3 and Figure 3. There were no statistically significant interaction effects between time after injury and group affiliations for any of the models. This means there were no significant differences in the degree of worsening or recovery from SWD or fatigue between the groups (mTBI, community controls, or trauma controls). In addition, there were no significant interaction effects between previous psychiatric disorders and group affiliation for any of the models. This means there were no differences in the effect of previous psychiatric disorders on SWD and fatigue between the groups (mTBI, community controls, or trauma controls). Therefore, the models were run without interaction effects. Age, sex, and previous history of psychiatric disorders were controlled for in all models. Across the groups, female sex was associated with increased sleep need (*p* = 0.014), poor sleep quality (*p* = 0.048), excessive daytime sleepiness (*p* = 0.013), and fatigue (*p* = 0.013). Older age was associated with fatigue (*p* = 0.048), and reporting a previous psychiatric disorder was associated with poor sleep quality (*p* < 0.001) and fatigue (*p* = 0.017).

**Table 3.** Mixed Logisitc Regression Models Comparing Patients with mTBI to Trauma Controls and Community Controls

Variables	Group difference mTBI vs. TC OR (95%CI)	*p*-value	Group difference mTBI vs. CC OR (95%CI)	*p*-value	Group difference TC vs. CC OR (95%CI)	*p*-value	Change over time OR (95%CI)	*p*-value
Increased sleep need	4.70 (1.73–12.76)	p = **0.002**	-	-	-	-		
2 weeks – 3 months							0.22 (0.12–0.40)	p**<0.001**
Poor Sleep Quality	2.96 (1.08–8.06)	p = **0.034**	7.30 (1.73–30.80)	p = **0.007**	4.71 (0.98–22.70)	p = 0.054		
2 weeks – 3 months							0.72 ( 0.43–1.21)	p = 0.217
2 weeks – 12 months							0.65 (0.38–1.10)	p = 0.109
Excessive Daytime Sleepiness	4.26 (1.01–18.07)	p = **0.049**	2.46 (0.61–9.92)	p = 0.205	0.81 (0.13–4.94)	p = 0.811		
2 weeks – 3 months							0.45 (0.20–1.06)	p = 0.054
2 weeks – 12 months							0.56 (0.25–1.22)	p = 0.144
Fatigue	7.38 (2.08–26.09)	p = **0.002**	7.22 (1.91–27.36)	p = **0.004**	1.45 (0.29–7.28)	p = 0.650		
2 weeks – 3 months							0.57 (0.30–1.08)	p = 0.086
2 weeks – 12 months							0.45 (0.23–0.88)	p = **0.020**

Mixed logisitc regression models without interaction between time x group affiliation and group x psychiatric history are presented. ORs in the models represents the increase in odds for having the symptoms depending on whether the participants belongs to the mTBI, TC or CC group. ORs over time represents the change in odds over time, and respective p-values indicate whether this change is statistically significant. A separate logistic regression model were completed, comparing patients with mild TBI, TC and CCs on poor sleep quality 2 weeks after injury, because this was the only time point were CCs had available data. Age, sex and prior psychiatric disorders were controlled for in all models.

mTBI: Mild Traumatic Brain Injury, TC: Trauma Controls, CC: Community Controls, OR: Odds Ratio. CI: 95% Confidence Interval. Bold p-values are significant at the 0.05 alpha level.

In the models, throughout the first year after injury, patients with mTBI had a significantly higher prevalence of problems with increased sleep need (odds ratio [OR], 4.7; confidence interval [CI], 1.7–12.8; *p* = 0.002), poor sleep quality (OR, 2.96; CI, 1.1–8.1; *p* = 0.034), excessive daytime sleepiness (OR, 4.26; CI, 1.01–18.07; *p* = 0.049), and fatigue (OR, 7.4; CI, 2.1–26.1; *p* = 0.002) compared to trauma controls.

Patients with mTBI also had a significantly higher prevalence than community controls with regard to poor sleep quality (OR, 7.3; CI, 17.0-38.8; *p* = 0.007) and fatigue (OR, 7.2; CI, 1.9–27.4; *p* = 0.004), but not excessive daytime sleepiness (OR, 2.5; CI, 0.6–9.9; *p* = 0.205). Trauma controls did not report significantly higher prevalence than community controls on problems with poor sleep quality (OR, 4.71; CI, 0.9–22.7; *p* = 0.054), excessive daytime sleepiness (OR, 0.81; CI, 0.13–4.90; *p* = 0.811), or fatigue (OR, 1.45; CI, 0.3–7.3; *p* = 0.650).

### The stability of poor sleep quality, excessive daytime sleepiness, and fatigue in mild traumatic brain injury and trauma controls

Fifty-two percent of patients with mTBI and 30% of trauma controls reported SWD or fatigue at least one time point after injury. Table 3 shows that there was a decrease (estimated decrease in odds) in prevalence of SWD and fatigue throughout the first year after injury, but significantly so only for increased sleep need between 2 weeks and 3 months (OR, 0.22; CI, 0.12–0.40; *p* < 0.001) and for fatigue between 2 weeks and 12 months after injury (OR, 0.45; CI, 0.23–0.88; *p* = 0.020). Poor sleep quality and excessive daytime sleepiness did not have a significant change in prevalence over time. Trauma controls did not display significantly different changes in SWD and fatigue over time compared to patients with mTBI. Given that patients with mTBI had higher levels of SWD and fatigue early after injury, this difference therefore remained similar across time points the first year after injury (Fig. 3).

In total, of the mTBI patients experiencing SWD and fatigue 2 weeks after injury (*n* = 136), approximately half (*n* = 72, 53%) continued to experience these problems for 3 months or longer. Of the trauma controls experiencing SWD and fatigue 2 weeks after injury (*n* = 20), 35% (*n* = 7) continued to experience these problems 3 months or longer.

### Prevalence of sleep-wake disturbance and fatigue after complicated mild traumatic brain injury

When comparing patients with complicated mTBI, patients with uncomplicated mTBI, and trauma controls, there were no significant interaction effects (group affiliation × time since injury or group affiliation × previous history of psychiatric disorders). The models were therefore run without interaction effects. Age, sex, and previous psychiatric disorders were controlled for in the models. Patients with complicated mTBI had statistically significant higher prevalence of problems with fatigue compared to patients with uncomplicated mTBI (OR, 3.6; CI, 1.0–12.3; *p* = 0.045). There were no statistically significant differences between patients with complicated and uncomplicated mTBI regarding levels of increased sleep need, poor sleep quality, or excessive daytime sleepiness (Table 4).

**Table 4.** Mixed Logistic Regression Models Comparing Patients with Complicated mTBI, Uncomplicated mTBI and Trauma Controls

Variables	Group difference cmTBI vs. umTBI OR (95%CI)	*p*-value	Group difference cmTBI vs. TC OR (95%CI)	*p*-value	Group difference umTBI vs. TC OR (95%CI)	*p*-value	Change over time OR (95%CI)	*p*-value
Increased sleep need	1.62 (0.59–4.40)	p = 0.347	7.20 (1.89–27.41)	p = **0.004**	4.45 (1.67–11.88)	p = **0.003**		
2 weeks – 3 months							0.24 (0.14–0.44)	p**<0.001**
Poor Sleep Quality	1.52 (0.45–5.18)	p = 0.500	4.17 (0.95–18.30)	p = 0.058	2.73 (1.01–7.41)	p = **0.047**		
2 weeks – 3 months							0.73 (0.43–1.24)	p = 0.248
2 weeks – 12 months							0.58 (0.33–1.02)	p = 0.058
Excessive Daytime Sleepiness	0.28 (0.37–2.07)	p = 0.211	1.46 (0.15–14.55)	p = 0.749	5.23 (1.24–22.08)	p = **0.024**		
2 weeks – 3 months							0.44 (0.19–0.99)	p = **0.048**
2 weeks – 12 months							0.60 (0.27–1.32)	p = 0.207
Fatigue	3.56 (1.03–12.29)	p = **0.045**	22.78 (4.17–124.20)	p<**0.001**	6.40 (1.82–22.42)	p = **0.004**		
2 weeks – 3 months							0.60 (0.32–1.15)	p = 0.123
2 weeks – 12 months							0.50 (0.25–0.97)	p = **0.041**

Mixed logistic regression models without interaction between time x group affiliation and psychiatric history x group affiliation are presented. ORs in the models represents the increase in odds for having the SWDs and fatigue depending on whether the participants belong to the complicated mTBI, uncomplicated mTBI or TC group. ORs over time represents the change in odds over time, and respective p-values indicate whether the change is statistically significant. Age, sex and prior psychiatric disorders were controlled for in all the models.

Bold p-values indicate significant p-values at the 0.05 alpha level.

cmTBI: Complicated Mild Traumatic Brain Injury, umTBI: Uncomplicated mTBI, TC: Trauma Controls, OR: Odds Ratio, CI: 95% Confidence Interval.

Patients with complicated mTBI had significantly higher prevalence of increased sleep need (OR, 7.2; CI, 1.9–27.4; *p* = 0.004) and fatigue (OR, 22.8; CI, 4.2–124.2; *p* < 0.001) compared to trauma controls. No significant differences were observed between patients with complicated mTBI and trauma controls in regard to poor sleep quality or excessive daytime sleepiness.

Patients with uncomplicated mTBI had significantly higher prevalence of increased sleep need (OR, 4.4; CI, 1.7–11.9; *p* = 0.003), poor sleep quality (OR, 2.7; CI, 1.0–7.4; *p* = 0.047), excessive daytime sleepiness (OR, 5.2; CI, 1.2–22.1; *p* = 0.024), and fatigue (OR; 6.4; CI, 1.8–22.4; *p* = 0.004) compared to trauma controls.

### Overlap of symptoms

**FIG. 4.**
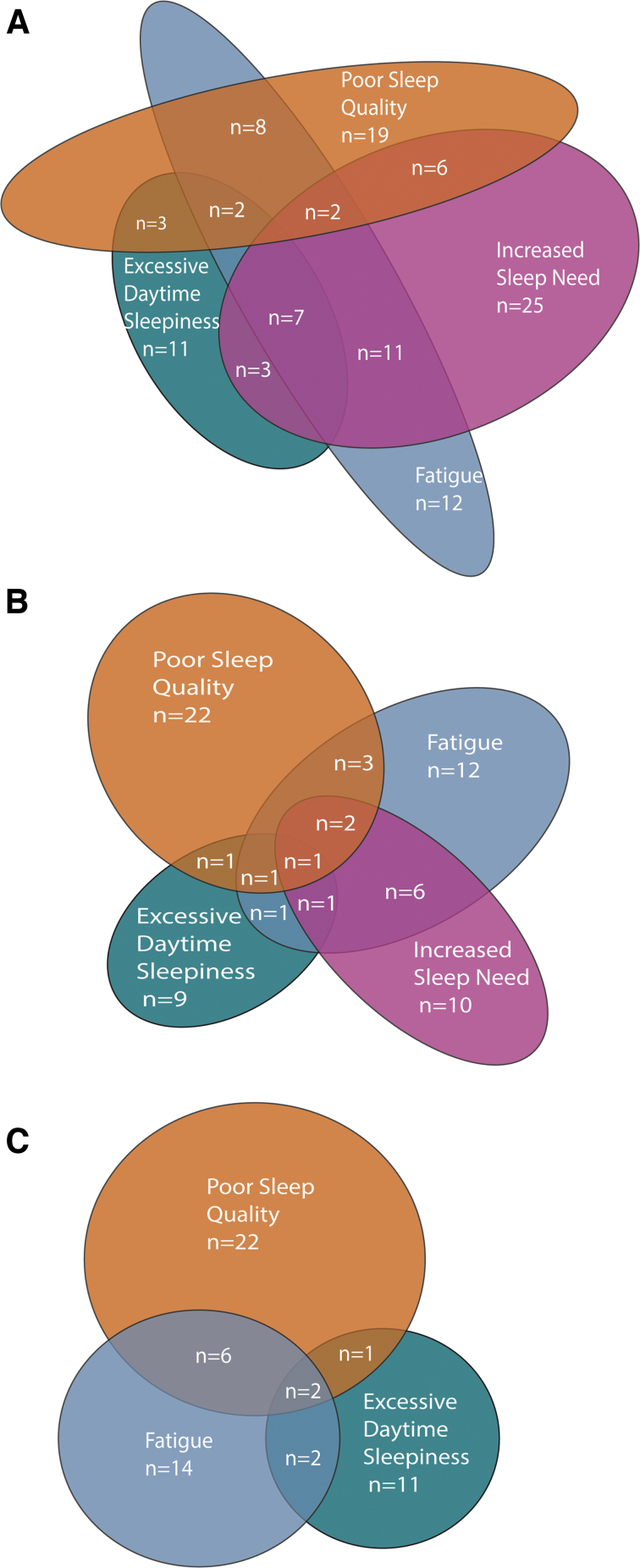
Overlap of increased sleep need, poor sleep quality, excessive daytime sleepiness, and fatigue 2 weeks to 12 months after injury for patients with mTBI. mTBI, mild traumatic brain injury; SWD, sleep-wake disturbance.

We examined the overlap between increased sleep need, poor sleep quality, excessive daytime sleepiness and fatigue. A considerably large proportion of patients with mTBI reported only one specific problem, rather than a combination of more problems at the same time point (Fig. 4). In total, 61%, 75%, and 79% of the patients with mTBI that reported problems at 2 weeks, 3 months, and 12 months, resepectively, reported one problem. Compared to SWDs, fewer patients reported fatigue as their only problem. Two weeks after injury, 29% of patients with mTBI reporting fatigue had fatigue as their only problem, whereas 45% reported increased sleep need, 46% reported poor sleep quality, and 41% reported excessive daytime sleepiness as their only problem. The proportion of patients with mTBI experiencing one problem rather than several problems increased over time. Three months after injury, 48% reported increased sleep need, 68% reported poor sleep quality, 57% reported excessive daytime sleepiness, and 43% reported fatigue as their only problem. By 12 months after injury, 71% reported poor sleep quality, 69% reported excessive daytime sleepiness, and 58% fatigue as their only problem (increased sleep need not included).

## Discussion

This study shows that SWD and fatigue are considerably more common throughout the first year after mTBI than after orthopedic trauma and in community controls. Our findings extend and substantiate previous studies by showing that *both trauma groups* (mTBI and orthopedic trauma controls) have a reduction of SWD and fatigue problems during the first year after injury; but whereas trauma controls return to levels similar to community controls 3 months after injury, a considerable proportion of patients with mTBI do not recover fully by 12 months after injury. In fact, as many as 53% of mTBI patients who experienced SWD or fatigue at 2 weeks after injury in the present study had persisting problems that lasted 3 months or longer. Stable symptoms lasting >3 months is a key criterion for insomnia and hypersomnolence disorder in the diagnostic manual DSM-5.^6^

These findings indicate that an mTBI is involved in onset and maintenance of SWD and fatigue, even when compared to other types of injuries, and when age, sex, and history of previous psychiatric disorders are controlled for. Patients with complicated mTBI had higher levels of fatigue than those with uncomplicated mTBI, which further suggests a dose-response relationship between brain injury severity and fatigue. Moreover, a few of the patients with mTBI had more than one problem with SWD and fatigue at once, indicated by the limited overlap between the individual measures of SWD and fatigue. This suggests that these different problems may be linked to different underlying mechanisms, and that personalized symptom-targeted strategies may be effective in the treatment of SWD and fatigue after mTBI.

Higher proportions of patients with mTBI had increased sleep need, poor sleep quality, excessive daytime sleepiness, and fatigue compared to trauma controls throughout the first year after injury. Compared to community controls, a larger proportion of patients with mTBI reported poor sleep quality and fatigue, whereas there was no statistically significant difference in excessive daytime sleepiness. Previous cross-sectional studies have indicated that both SWD and fatigue are more prevalent within the first month after mTBI, compared to trauma controls^47^ and community controls.^30,48–50^ Other longitudinal studies with control groups have also shown that SWD and fatigue are more common the first month after injury in patients with mTBI than in trauma controls^24,25,51^ and community controls.^51^ However, in these studies, patients with mTBI have typically recovered to similar levels of SWD and fatigue as trauma-^23–25^ and community controls^51^ 1–3 months after injury.

Previous findings therefore imply that patients with mTBI recover more rapidly than trauma controls. In contrast, we did not observe any statistically significant interaction effects between group (mTBI vs. trauma controls) and time in our study. This means that the two injury groups did not differ in rate of recovery from SWD or fatigue. In fact, the present study and longitudinal studies without control groups^1,10^ show that a substantial number of patients with mTBI report SWD and fatigue also >3 months after injury. We also observed that trauma controls had recovered to similar levels of SWD and fatigue as community controls at 3 months after injury. Earlier longitudinal studies also report higher rates of SWD at 6, 12, and 18 months after mTBI^1^ compared to control groups^37,38^ and the general population.^52^ In the context of the present results, these findings support the hypothesis that the brain injury itself may be involved in onset of SWD and fatigue.

We did not have direct information about shift work, social jet lag, sleep satiation, or rhythms, which may also impact sleep-wake outcomes.^53,54^ However, the groups included in our study did not differ much in the distribution of occupational status or in their self-reported average total sleep time the last 2 weeks before inclusion to the study (i.e., before the injury for the patient groups). Neither was there any evidence of a difference between patients with mTBI and trauma controls regarding return to work/school after injury. Although not providing the same stringent level of control as in other studies,^37,38^ there were therefore no obvious contextual factors indicating that there should be any substantial differences in sleep habits or rhythms across the groups.

The proportion of patients that was injured because of violence was higher in the mTBI group than in the trauma control group. Earlier studies on mTBI have found that injuries caused by violence are associated with more time away from work,^55^ post-traumatic stress, and depression.^56^ More generally, violence is often associated with stress-related symptoms, including problems with SWD and fatigue.^57^ Consequently, we cannot completely rule out that differences in injury mechanisms may have contributed to the differences in SWD and fatigue between patients with mTBI and trauma controls.

Pain can be associated with SWD and fatigue after any kind of trauma.^22^ In our study, both patient groups reported higher levels of pain intensity than community controls, as well as considerably higher levels of pain intensity at 2 weeks than at 3 and 12 months after injury. As expected, the mTBI group mainly reported pain in their head, face, or neck region, whereas trauma controls and community controls predominantly reported pain in other parts of their body. Despite this difference in pain topography, level of pain intensity did not differ between the mTBI group and trauma controls at any time point. It is therefore unlikely that the differences in SWD between these groups can be explained by differences in pain intensity.

In the present study design,^32^ we applied more liberal inclusion criteria compared to previous longitudinal studies comparing mTBI patients with control groups on SWD and fatigue measures.^23–25^ Differences in exclusion criteria may explain the aforementioned discrepancies between the present study and earlier controlled longitudinal studies investigating SWD and fatigue after mTBI.^23–25^ These earlier studies excluded many patients, such as patients with complicated mTBI,^25^ or patients with pre-existing conditions, such as psychiatric problems^23,24^ or previous mTBI.^23,24^ Only patients with *previous complicated*, but not *uncomplicated*, mTBI were excluded from participation in our study. Patients with *current* complicated mTBI were included in our study, albeit these patients accounted for a small fraction of the total sample (9% with intracranial findings on CT or MRI). Regarding previous psychiatric problems, only patients with mTBI and *severe* ongoing psychiatric disorders that would complicate follow-up were excluded in the present study. Reporting a previous psychiatric disorder was generally associated with poor sleep quality and fatigue across groups, but there was no evidence that this relationship differed between patients with mTBI and the control groups.

Importantly, even when controlling for previous psychiatric disorders in our analyses, we obtained different results than other studies.^23–25^ Two of the aforementioned studies^23,24^ stated that the aim of their study was to have a more homogeneous sample, whereas the present study aimed for a representative (and, as a consequence, more heterogeneous) mTBI sample. Another difference between the present study and earlier controlled longitudinal studies is that we also included patients from the primary healthcare setting, persons who are not often included in mTBI research. A recent population-based longitudinal study that applied similar exclusion procedures as the present study, and also included patients in a primary healthcare system, similarly found SWD to remain a significant long-term problem after mTBI.^1^ Altogether, these findings show that when including patients with mTBI who are often not included in mTBI research, SWD and fatigue appear to be considerable long-term problems after mTBI.

Prevalence of fatigue problems was statistically significantly higher in the subgroup of patients with complicated mTBI compared to the group considered to have uncomplicated mTBI. Although patients with uncomplicated mTBI still had significantly higher prevalence of fatigue compared to trauma controls, this further supports a link between visible brain pathology and fatigue. We failed to demonstrate any statistically significant differences in SWD between the complicated and uncomplicated mTBI groups; however, the results from the subgroup analyses should be interpreted with caution considering the modest sample size and relatively large confidence intervals in these analyses. Earlier studies have shown that complicated mTBI may be associated with worse outcomes and higher levels of symptoms.^58–60^ There are, however, only a few existing large-scale longitudinal studies, and these show small to no effects of findings on early MRI and CT.^61^ Consequently, no consensus has been reached regarding the predictive value of complicated mTBI on symptom development post-mTBI.^62^

The present study is the first study to show a relationship between complicated mTBI and fatigue after adult mTBI. These findings indicate that the effects of the brain injury itself may be involved in the onset of fatigue, which has previously been demonstrated only after moderate and severe TBI.^63^ The imaging protocol used in the present study was comprehensive and state-of-the-art from a clinical perspective. It is possible that some patients with mTBI in our sample have poor white matter organization attributable to their mTBI, which is only detectable with potentially more-sensitive MRI techniques, such as diffusion tensor imaging (DTI).^64^ Earlier studies using DTI and DWI have indicated a link between disrupted white matter organization and SWD and fatigue after mTBI, but with small to modest sample sizes and varying time after injury.^65–67^ Our future work will further investigate *longitudinal* associations between white matter organization and the development of SWD after mTBI using advanced MRI techniques, including DTI, DWI, and structural morphological measures.

It is important to note that most mTBI patients (55%) and trauma controls (77%) did not report any SWD or fatigue during the first year after injury. We also observed a decrease in prevalence of SWD and fatigue throughout the first year after injury, statistically significant so for increased sleep need and fatigue. Still, 55% of patients with mTBI who had SWD and fatigue 2 weeks after injury had problems that lasted 3 months or longer. Detection of SWD and fatigue is crucial for optimal recovery in patients with mTBI, because such problems are related to a range of negative consequences, including impeded long-term recovery,^68^ reduced cognitive functioning,^1,69^ social and functional outcome,^1,70^ emotional processing,^71^ and other post-concussive symptoms.^1^ Screening for SWD and fatigue in an early phase can therefore be beneficial for these patients because interventions and advice about sleep hygiene can be effective and improve overall outcome.^5^ On the other hand, given that approximately half of patients with SWD and fatigue recovered, future work should also focus on more detailed delineation of risk factors for prolonged SWD and fatigue as well as protective factors for recovery.

We observed limited overlap between individual symptom measures of SWD and fatigue across all time points in this study (Fig. 4). For patients with mTBI who reported SWD or fatigue, 61%, 75%, and 79% reported experiencing one problem rather than two or more problems at 2 weeks, 3 months, and 12 months, respectively. That relatively few patients with mTBI reported fatigue as their only problem 2 weeks after injury is somewhat mirroring the diagnostic criteria for insomnia and hypersomnolence disorder, in which these disorders often are accompanied by daytime distress in the form of fatigue.^6^ All SWDs and fatigue decreased over time, resulting in reduced overlap, but the number of patients with mTBI experiencing one problem remained stable, resulting in an increasing proportion of patients with mTBI experiencing one problem with time.

The present study therefore indicates that it is important to include several measures to capture the full extent of SWD and fatigue after mTBI. Moreover, limited overlap between increased sleep need, poor sleep quality, excessive daytime sleepiness, and fatigue suggests that these problems may have different underlying mechanisms.^31^ If these problems have distinct underlying mechanisms, personalized^72^ and symptom-targeted treatment strategies may be more viable than a generic approach aimed at post-concussive problems in general.^73^

Altogether, findings from the present study show that persons with mTBI can have different paths to the development and maintenance or recovery from SWD and fatigue. Clinical decision making is challenging after mTBI, but sleep is a modifiable factor.^73^ Targeted SWD interventions have been shown to be effective in reducing the symptom burden of comorbid symptoms in other patient populations,^74^ and preliminary findings are also promising for patients with mTBI.^75^

### Limitations

Our results rely on self-reported data collected through structured interviews and questionnaires. It is important to capture the person's own perceived state,^7^ but questionnaires may not be as precise in capturing clinically relevant problems as clinical diagnostic interviews.^6^ Objective measures, such as polysomnography and actigraphy, may also be effective in measuring total sleep time or sleep efficiency.^38,76^ Insomnia is based on the subjective assessment of having disturbed sleep,^6^ whereas pleiosomnia is based on objective assessments of sleep quantity and/or daytime sleepiness.^36–38^ This distinction is important given that earlier studies have revealed important differences between objective and subjective sleep measures in TBI. Both subjective sleep duration and subjective sleepiness may be underestimated relative to objective assessments of sleep duration and sleepiness in TBI.^37,38^ Consequently, our finding that subjective sleepiness was not more prevalent after mTBI than in community controls should be interpreted with caution given that there may be differences in objective sleepiness.

We had a high success rate in contacting participants for interviews, but less success in receiving complete questionnaires from our participants. Based on our attrition analyses, we found treating the data as missing at random to be reasonable, and our primary analysis is robust to missing data under the missing at random assumption.^77^ Therefore, the missing data are not expected to have a large impact on our results. We did not investigate other SWDs, such as circadian rhythm sleep disorder^78^ or obstructive sleep apnea,^79^ which can affect increased sleep need, sleep quality, excessive daytime sleepiness, or fatigue.^17^

We applied relatively conservative cut-off scores for determining SWD and fatigue problems, also compared to earlier similar studies.^1,10^ Currently, there is no consensus in the literature on whether to use originally defined cutoffs^1^ or study-specific cutoffs.^10^ The use of different cut-off values limits direct comparisons between studies. However, a strength in our approach was that our cut-off values were based on recommendations from earlier validation studies^44^ and studies comparing subjective and objective measures.^42^ Using strict cut-off values increased the chance of false negative findings, but in the context of our study, we are at least confident that we have avoided an overestimation of significant problems with SWD and fatigue.^44^

Fatigue, as measured with the FSS, may overlap with conditions such as depression and apathy.^80^ We adjusted for previous psychiatric disorders in our analyses, but we cannot rule out that the levels of fatigue measured in our study may be overlapping or interacting with other post-injury symptomatology that was not included in our analyses.

There is a substantial pre-clinical and clinical body of evidence that repeated mTBI has a more severe effect on SWD than a single mTBI.^81–84^ Our data on previous uncomplicated mTBI was based on self-report and obtained according to procedures used in other studies.^35^ However, considering the uncertainty of such data,^85^ and the considerable variance in time after injury (Table 1), we did not consider our data to have sufficient quality for being included in formal analyses.

The findings in the present study were limited in the comparison of patients with mTBI to community controls because community controls did not provide data at all the time points. We did not observe much change in scores for community controls on SWD and fatigue measures that were available for two time points. It is therefore not likely that this lack of data has significant impact on the main conclusions in this study.

One strength of the present study was the inclusion of both trauma controls and community controls. The sample size in the control groups was lower than in the mTBI group because of the lower expected variance in symptoms in the former groups. No formal *a priori* power estimation was performed for determining the sample size for the specific analyses included this study, but the sample size of control groups are comparable to,^25^ or larger than,^23,24,37,38^ in other studies reporting effects on SWD and fatigue after mTBI.

## Conclusions

The findings in this study demonstrate that mTBI is associated with the presence and maintenance of SWD and fatigue over time. Problems with SWD and fatigue resolve for most, but persist and become chronic for a considerable subgroup of patients with mTBI. It was more common for patients with mTBI to experience one problem with SWD or fatigue, rather than several problems. Moreover, patients with complicated mTBI had higher prevalence of fatigue than did patients with uncomplicated mTBI. Different SWDs and fatigue may therefore have different origins and underlying mechanisms after mTBI. These results therefore indicate that patients with mTBI may benefit from personalized and targeted treatment strategies aimed at the patient's specific symptom burden.
